# Aberrant neural computation of social controllability in nicotine-dependent humans

**DOI:** 10.21203/rs.3.rs-3854519/v1

**Published:** 2024-01-24

**Authors:** Xiaosi Gu, Caroline McLaughlin, Qixiu Fu, Soojung Na, Matthew Heflin, Vincenzo Fiore

**Affiliations:** Icahn School of Medicine at Mount Sinai

**Keywords:** social controllability, nicotine addiction, functional magnetic resonance imaging (fMRI), computational psychiatry, vmPFC, midbrain

## Abstract

Social controllability, defined as the ability to exert influence when interacting with others, is crucial for optimal decision-making. Inability to do so might contribute to maladaptive behaviors such as drug use, which often takes place in social settings. Here, we examined nicotine-dependent humans using fMRI, as they made choices that could influence the proposals from simulated partners. Computational modeling revealed that smokers under-estimated the influence of their actions and self-reported a reduced sense of control, compared to non-smokers. These findings were replicated in a large independent sample of participants recruited online. Neurally, smokers showed reduced tracking of forward projected choice values in the ventromedial prefrontal cortex, and impaired computation of social prediction errors in the midbrain. These results demonstrate that smokers were less accurate in estimating their personal influence when the social environment calls for control, providing a neurocomputational account for the social cognitive deficits in this population.

## Introduction

The environment we live in is highly complex and uncertain. As such, one must be able to exert control over the environment to achieve desired outcomes and avoid unwanted ones. For humans, our social environment might present the most challenging situation for exerting behavioral control, due to its high degree of complexity. *Social controllability,* defined as one’s ability to exert control during interpersonal interactions, is thus essential for optimal decision-making in everyday scenarios ^1^. The breakdown of this process might lead to suboptimal behaviors such as substance abuse, which often takes place with other people or under the influence of other people. Smoking and nicotine use, for example, is a highly social behavior, especially in younger adults ^2,3^. While prior work has examined many constructs related to smoking (e.g. cue reactivity, impulsive control), little is known regarding the mechanisms underlying social cognitive deficits associated with this population. Specially, it remains elusive how human smokers exert and perceive social control differently from non-smokers at both neural and computational levels.

Previously, reinforcement learning (RL) algorithms have been used to capture how drugs might alter neural computations of decision variables, such as encoding of reward prediction errors by the mesolimbic circuit ^4-6^. Furthermore, economic preference models such as temporal discounting have also revealed that substance-dependent individuals show a preference for smaller immediate rewards over delayed larger rewards ^7-9^ which may reflect a complex interaction between time perception and risk preference ^10-12^. More recent computational models have linked addiction to dysfunctions in model-based control ^13,14^ and forward planning ^15-17^. Others have postulated that these model-based planning deficits are further amplified by complex environments ^18,19^. However, empirical evidence from substance-dependent humans supporting these computational frameworks – especially in the context of social decision-making - is still scarce. Here, we aim to directly examine the neural computations underlying social controllability in substance-dependent humans, using a computational psychiatry approach and nicotine addiction as a test case.

**Figure 1.**
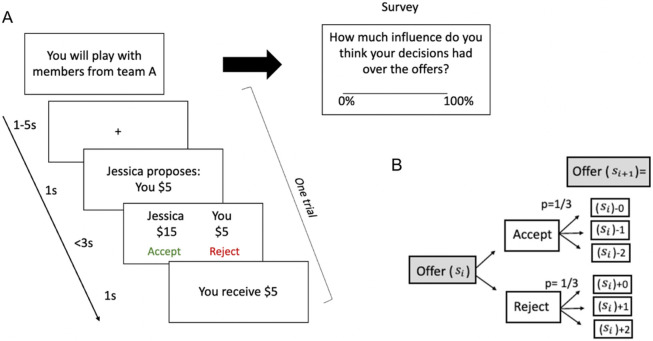
Experimental paradigm. A) Participants played the social controllability task. At the start of the task, participants were only informed about the team with whom they were playing but no how the teams differed. As such, they would need to learn the contingencies between their actions and consequences during the task. Within the same block, they played with different individuals from the same team for each trial. Participants’ main task is to decide whether to accept or reject an offer from a virtual team member proposing how to divide $20. At the end of the game, participants rated their perceived controllability over their interactions. B) A schematic of contingencies for the controllable condition of the task displays how the following offer was generated based on the participant’s previous action. If participants accepted the current offer ($s_{i}$), the subsequent offer ($s_{k+1}$) decreased by $0, $1, $2 with a 1/3 probability for each. If they rejected the current offer ($s_{i}$), the subsequent offer ($s_{k+1}$) increased by $0, $1, $2 with a 1/3 probability for each.

Based on the literature reviewed thus far, we hypothesized that smokers would demonstrate reduced ability to exert social control, subserved by reduced neural computations of social forward planning and learning signals. At the neural level, previous work shows that the ventromedial prefrontal cortex (vmPFC) is important for tracking the downstream effects of agents’ current choices in order to exploit the controllability of a simulated social environment in healthy volunteers ^1^. The vmPFC has been consistently shown to encode cognitive maps, an efficient way to represent task space and environmental structure that are crucial for model-based planning ^20^. Using a similar decision-making task (Fig. 1) in smokers and non-smokers across two independent samples (in-person fMRI sample: n = 17 for smokers and n = 25 for non-smokers; online replication sample: n = 72 for smokers and n = 147 for non-smokers; see Methods and **Tables S1-S2** for participant characteristics), the current study examined how vmPFC-dependent social controllability computation might differ between nicotine-dependent humans and non-using controls. Participants made choices about accepting or rejecting an monetary proposal from stimulated partners (i.e. the ultimatum game; Fig. 1A); crucially and different from a typical ultimatum game, their choices could increase or decrease the future monetary proposals from the partners in a probabilistic fashion (Fig. 1B). We used computational modeling (see Methods) to quantify a key parameter δ (“estimated influence”) representing the mentally simulated influence of one’s actions on future social outcomes ^1^. We predicted that smokers would under-estimate the level of influence their actions have on the future, compared to controls, accompanied by reduced neural activation in the vmPFC. A secondary analysis will also examine neural activations (e.g. midbrain) related to social prediction errors in both groups.

## Results

### Smokers failed to exploit the controllability of their social interactions

**Figure 2.**
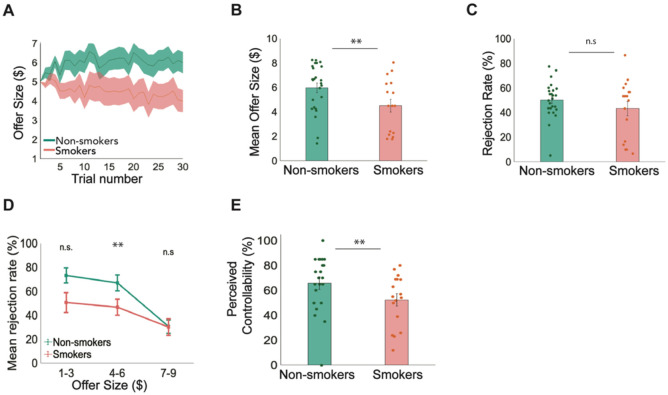
Smokers failed to exploit the controllability of their environment compared to non-smokers (in-person sample). In the controllable condition of the task, **A)** smokers’ offer sizes slightly decrease from trial to trial while non-smokers' offer sizes increase from trial to trial. **B)** A two-sampled t-test revealed that individual mean offer sizes are significantly lower for smokers ($4.5±0.52) compared to non-smokers ($5.98±0.39; t(40)= 2.31, p= 0.0131). **C)** Overall rejection rate was not significantly different for smokers (43.23%±5.8) compared to non-smokers (50.26%±3.0; p>0.05). **D)** However, when rejection rates were divided and categorized by low ($l-$3), medium ($4-$6) and high ($7-$9) offers, smokers had a significantly lower rejection rate for medium offer sizes (46.72%±6.71) compared to non-smokers (66.93%±6.64; t(40)= 2.27, p=0.0144). **E)** Perceived controllability rated on a scale of 1% to 100% after each condition of the task was significantly lower for smokers (52.40%±5.04) compared to non-smokers (65.91%±5.06; t(40)=1.93, p= 0.031).

We first evaluated model-agnostic measures of subjects’ behaviors to determine if they were able to detect and exploit the controllability of the interactions in this task, indexed by the offer amount they were able to obtain. For non-smokers, we found that they successfully raised the offers over time (Fig. 2A). In contrast, smokers were unable to exploit the controllability of their interactions as indicated by the flat or even slight decrease in offer sizes over time (Fig. 2B). On average, smokers received lower offers on average ($4.5 ± 0.52) compared to controls ($5.98 ± 0.39; t(40) = 2.31, p = 0.0131;Figure 2B). This suggests that overall, smokers failed to exploit the controllability of the interactions.

Given the contingencies designed in the game, participants would need to reject and forgo smaller offers to strategically raise future offers. Thus, we compared rejection rates between smokers and non-smokers. We found that total rejection rates were not significantly different between smokers (43.23%±5.8) and non-smokers (50.26%±3.0) (Fig. 2C). However, when offers were grouped into low ($1-$3), medium ($4-$6) and high ($7-$9), smokers’ rejection rates of medium sized offers (46.72%±6.71) were lower than those of non-smokers (66.93%±6.64; t(40) = 2.27, p = 0.0144; Fig. 2D). This result suggests that smokers did not use “strategic rejection” as well as non-smokers, which contributed to their inability to raise offers overall. In parallel to their choice behaviors, smokers also reported a lower sense of control (52.40%±5.04) compared to non-smokers (50.26%±3.0; t(40) = 1.93, p = 0.031; Fig. 2E). Taken together, these model-agnostic behavioral results reveal that nicotine-smokers failed to exploit the controllability of the social environment.

### Smokers under-estimated the future influence of their current choices

**Figure 3.**
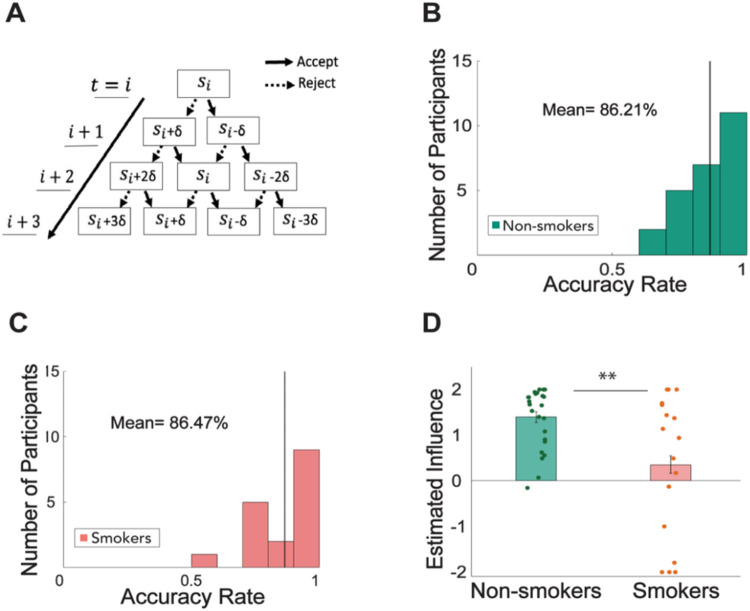
A computational model of forward thinking (FT) reveled that smokers were able to mentally simulate future interactions, but inaccurately under-estimated their influence on future offers (in-person sample). **A)** A schematic demonstrating how an agent might mentally simulate the values of future states using a forward-thinking model. Simulated offers increase or decrease by estimated influence δ, dependent on participants’ choice to accept or reject the split of money. **B)** The 2-step model of FT predicted non-smokers’ choice in the ‘Controllable’ condition of the task with a mean accuracy rate of 86.21% (bold black line). **C)** The 2-step model of FT predicted smokers’ choice in the ‘Controllable’ condition of the task with a mean accuracy rate of 86.47%. **D)** The parameter of interest, estimated influence, estimated from the 2-step FT model in the ‘Controllable’ condition of the task was significantly lower for nicotine-smokers (0.352±1.54) compared to non-smokers (1.40±0.654; t(40)= −3.02, p= 0.002).

Next, we sought to uncover the computational mechanisms underlying subjects’ choices using a series of models involving various depths of future steps computations (1 to 4), not involving future thinking (but still considered aversion to norm violation), or only using cached value in a model-free fashion without forward thinking or norm violation. Model comparison results demonstrated that in the controllable condition, all the FT models better explained both smokers’ and non-smokers’ choices compared to the 0-step model or model-free reinforcement learning model (**Table S3**). Consistent with our previous work, the 2-step FT model also showed good parameter recoverability (**Table S4-S5**) and was selected for subsequent statistical and neural analyses. Overall, the 2-step FT model predicted non-smokers’ choices with an 86.21% accuracy (Fig. 3B) and smokers’ choices with an 86.47% accuracy (Fig. 3C).

Next, we examined parameters from the 2-step model (see Table 1 for all parameter values). Our key parameter of interest here is δ, which represents the mentally estimated controllability or influence of one’s current choices on future offers. We found a significant difference in this parameter between smokers (0.352 ± 1.54) and non-smokers (1.40 ± 0.654; t(40)= −3.02, p = 0.002; Fig. 3D). This result suggests that while engaging a 2-step forward-thinking model, smokers significantly under-estimated how much their current choices might affect future interactions compared to non-smokers. Interestingly, no other parameters showed a significant difference between groups. These findings suggest that a lower δ – or reduced estimate of influence of one’s actions on the environment – explains the model-agnostic finding of smokers receiving a lower level of offer overall.

### Replication of behavioral and computational results in the online sample

**Figure 4.**
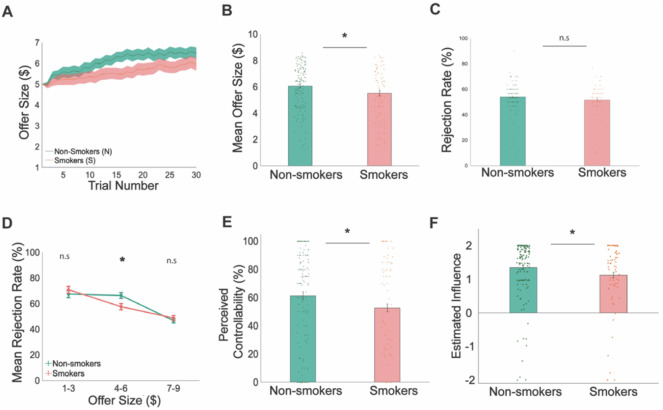
Replication study: smokers perceived and inaccurately under-estimated the influence of their current choices on future interactions in an independent online sample. In the controllable condition of the task **A)** smokers’ offers increased trial-by-trial but remained below non-smokers’ offer sizes. **B)** A non-parametric test shows that mean offer sizes were significant lower for smokers ($5.53±1.85) compared to non-smokers ($6.06±1.68; p= 0.0266). **C)** Overall rejection rates were not significantly different between smokers (51.57±0.12) and non-smokers (53.97%±0.10; p>0.05). **D)** However, when rejection rates were divided and categorized by low ($1-$3), medium ($4-$6) and high ($7-$9) offers, a non-parametric bootstrapping test shows that smokers had a significantly lower rejection rate for medium offer sizes (57.59%±0.29) compared to non-smokers (66.40%±0.27; p=0.0175). **E)** Perceived controllability rated on a scale of 1% to 100% after each condition of the task was significantly lower among smokers (52.68%) compared to non-smokers (61.32; p= 0.0442). **F)** The parameter of interest, estimated influence, estimated from the 2-step forward thinking model in the ‘Controllable’ condition of the task, was significantly lower for smokers (1.119±1.0164) compared to non-smokers (1.351±0.8334; p = 0.0447).

Next, we analyzed data collected from our large online sample to examine if the behavioral and computational findings from the in-person study were generalizable to a group of smokers with less severe nicotine dependence (**Table S2**). In line with findings from the in-person sample, smokers recruited online also had reduced offer sizes over time compared to non-smokers, although their offer trajectory showed a slight upward trend (Fig. 4A). On average, smokers still had significantly lower mean offer size (5.53 ± 1.85) compared to non-smokers (6.06 ± 1.68; bootstrapping p = 0.0266; Fig. 4B), albeit a smaller group difference. These results demonstrated that smokers recruited online were also less successful than non-smokers in exploiting the controllability of the social environment.

Consistent with our in-person sample, we found that total rejection rates did not differ significantly between smokers (51.57%±0.12) and non-smokers (53.97%±0.10) (p = 0.0770; Fig. 4C). When we analyzed rejection rates based on offer size (low: $1-$3, medium: $4-$6, and high: $7-$9), we replicated the previously observed pattern of lower rejection rates among smokers for medium offers (smokers: 57.59%±0.29, non-smokers: 66.40 ± 0.27; p = 0.0175; Fig. 4D). Online smokers also self-reported a reduced sense of control (smokers: 52.68%±34.46, non-smokers: 61.32%±34.63; p = 0.0442; Fig. 4E), similar to what we observed in the in-person sample.

Finally, we applied the same computational models to fit the choice data collected from online participants. Overall, model-based results were also consistent between the in-person and online sample. Specifically, we found that the estimated influence parameter from the 2-step model was significantly reduced in smokers (1.12 ± 1.02) compared to non-smokers (1.35 ± 0.83; p-value = 0.0447; Fig. 4F). Collectively, the larger online sample replicated key behavioral and computational findings from the in-person study, further confirming aberrant forward-thinking in smokers across a wide range of severity.

### Smokers showed aberrant encoding of forward thinking value in the vmPFC

**Figure 5.**
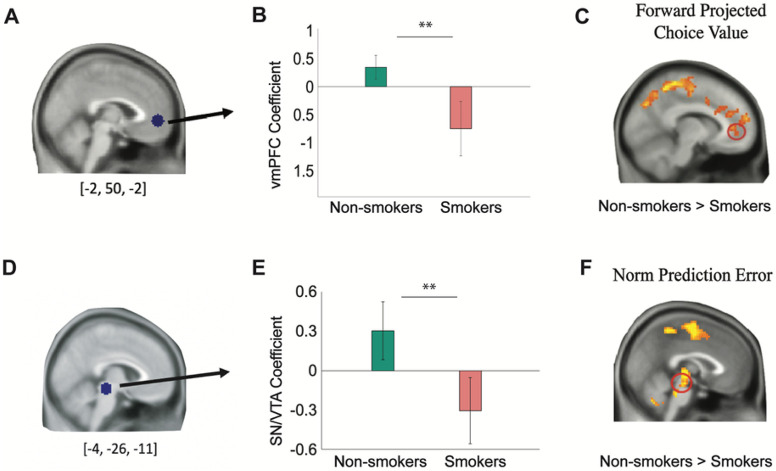
Smokers show aberrant ventromedial prefrontal cortex (vmPFC) and midbrain activity while computing forward projected choice values and norm prediction errors, respectively. **A)** vmPFC ROI coefficient [−2, 50, −2] was selected from an independent study ^21^ and extracted from an 8-mm radius sphere for estimated choice values from the 2-step forward thinking model in the controllable condition of the task ($P_{\text{FDR}}<0.05$, k>50). **B)** vmPFC coefficients were significantly greater for non-smokers (0.347±0.211) compared to smokers (−0.749±0.486; t(40)= −2.31, p=0,013. **C)** One-way between-subject ANOVA test for the whole-brain map further revealed that BOLD responses in the vmPFC is greater for non-smokers compared to smokers ($P_{FDR}<0.05$ and k>50). **D)** The midbrain ROI coefficient [−4, −26, −11], covering the substantia nigra (SN) and ventral tegmental area (VTA), were selected from an independent study ^25^ and extracted for norm prediction errors from the 2-step forward thinking model in the controllable condition of the task. **E)** SN/VTA coefficients were significantly greater for non-smokers (0.302±0.220) compared to smokers (−0.306±0.252; t(40)= −1.80, p=0.040). **F)** One-way between-subject ANOVA test for the whole-brain map further revealed that neural responses to norm prediction errors in the midbrain was greater for non-smokers compared to smokers ($P_{FDR}<0.05$ and k>50).

For the neural analyses, our primary interest was to examine neural activities associated with the FT value signal during forward thinking, which was found to be encoded by the vmPFC in healthy volunteers ^1^. Thus, we first conducted ROI analysis using beta coefficients extracted from an independent ROI of the vmPFC [−2, 50, −2] ^21^ (Fig. 5A). This analysis revealed that vmPFC activations related to total choice value were significantly greater for non-smokers (parameter estimate: 0.347 ± 0.211) compared to smokers (parameter estimate: −0.749 ±0.486; two-sample t(40) = −2.31, p = 0.013; Fig. 5B). Whole-brain analysis (Fig. 5C) further confirmed that even after whole-brain correction, BOLD responses in the vmPFC were still significantly greater for non-smokers compared to smokers (P_FDR_<0.05, k > 50). Overall, these results indicate aberrant neural encoding for the computation of FT values in the vmPFC in smokers.

### Reduced activation to norm prediction errors in the midbrain in smokers compared to non-smokers

We additionally evaluated nPE encoding, given that smokers have previously demonstrated altered learning ^22^ and that nPEs were an important learning signal driving norm updating in this game. Based on previous research ^23,24^ demonstrating the involvement of mesolimbic structures (e.g. midbrain) in reward-based learning, we extracted neural signals tracking nPE using an independent ROI of midbrain [4, −26, −11] (Fig. 5D) that included regions of the ventral tegmental area and substantia nigra ^25^. We found that while nPEs positively scaled with midbrain activity in non-smokers (parameter estimate: 0.302 ± 0.220), this relationship was inversed in smokers (parameter estimate: −0.306 ± 0.252; two-sample t(40)= −1.80, p = 0.040; Fig. 5E). Whole brain analysis further confirmed this significant group difference in that smokers showed reduced activation than non-smokers in midbrain activity related to nPEs (Fig. 5F; $P_{FDR}<0.05$ and k>50). Collectively, these results are consistent with previous findings indicating prediction error encoding deficits in smokers and expand beyond previous findings by showing how nicotine addiction is also associated to aberrant updating of information in the context of dynamic social interactions ^26-28^.

## Discussion

Social controllability, the ability to exert control during social interactions, is crucial for behavioral adaptability. Previous research suggests that accurately simulating the impact of one’s actions on future states is crucial for exerting social control, a process subserved by a vmPFC circuit ^29,30^. Here, we demonstrated how neural computation of social control might be altered in nicotine addiction. Our main finding demonstrated that, in a controllable social environment, smokers under-estimated the downstream influence of their current choices and thus, failed to exploit the controllability of their social interactions. These findings were consistent in a larger online sample, further confirming the observed effect among smokers. Neurally, smokers showed reduced encoding of forward-thinking values in the vmPFC and reduced tracking of norm prediction errors in the midbrain. Collectively, these results suggest that social cognitive deficits in addiction might be associated with complex decision processes involving future-oriented thinking.

Previous work has often focused on how individuals with SUDs exert control over motor impulses or over actions with immediate outcomes. These studies have demonstrated reduced cognitive control and high impulsivity levels in these individuals ^31-34^. Based on this literature, one might expect that smokers would exhibit reduced planning horizon as suggested by previous computational work ^6,35^. Here, formal model comparison showed that smokers engaged a similar 2-step forward thinking model as non-smoking controls, yet under-estimated the influence of their actions on future states (lower δ value) compared to non-smokers. This interpretation is consistent with the participants’ subjective assessment of controllability in the game, where smokers indicated they perceived future offers as being less affected by their actions, compared to matching controls. These findings echo with the idea that it is maladaptive for an agent to infer that they have less impact on the future than they actually do, as one might not only miss out exploitable opportunities but also fail to avoid negative consequences in the future ^36^. Although no punishment was introduced as outcome in our study design, our work provides a computational framework and paradigm that could be used by future research to examine mental simulation of future negative consequences. It also remains to be investigated whether the findings of smokers’ under-estimation of their influence is associated with a mismatch between available cognitive resources and environmental complexity ^18,19^.

Our finding is also in line with and provides a computational explanation for findings of increased discounting rates of future rewards associated with SUD ^7-9^ and that simulation of future events reduces delay discounting and cigarette consumption ^37^. Importantly, our study expands this literature by showing that future-oriented valuation of one’s own agency (i.e. calculating the impact of one’s action on future events) is altered in smokers and might become a key factor in their altered estimation of future values, contributing to the temporal discounting effect. This could introduce new avenues for intervention, as an accurate mental representation of how current actions impact downstream outcomes may help individuals with SUD reevaluate drug-related choices.

The cognitive deficit in forward thinking observed in smokers was corroborated by our neural finding of reduced activity in the vmPFC in tracking projected total choice value in this group. The vmPFC has been heavily implicated in both addiction ^38-40^ and value-based decision-making ^1,27,41,42^. Specifically, decreased activity of the vmPFC has been associated with a reduced preference for delayed rewards and impairments in valuation processes ^43-45^. In both occasional and nicotine-dependent smokers, activity in the mPFC is associated with decisions to purchase or consume cigarettes ^46^. Here, we observed that vmPFC activity was in fact anticorrelated with projected total values in smokers, deviating from the positive association between the two measures observed in non-smokers. This finding, along with past research, demonstrates that deficit in the vmPFC is associated with suboptimal decision-making in nicotine addiction. Our result also expands beyond previous work by demonstrating that aberrant neural activity in the vmPFC is detrimental to not only value representation but also future-oriented, model-based planning. This finding is consistent with more recent work highlighting the role of vmPFC in presenting states and task structure ^20,47^.

We also found aberrant neural tracking of norm prediction errors in the midbrain in smokers. Norm prediction errors, defined as the difference between the actual social signal (i.e. proposed offer) and one’s expectation (i.e. internal norms), allows an agent to flexibly adapt to a changing social environment. Previously, activities in midbrain structures – including the substantia nigra and ventral tegmental area-have been linked to social norm updating and decision-making during the ultimatum game ^48^. Existing work has also indicated altered non-social reward prediction error encoding in individuals with addiction ^28,49^. Hence, we extend both lines of previous work by demonstrating that addiction is also linked to neural deficits in the midbrain during aberrant norm updating in complex social environments.

Finally, despite the acknowledgment of the importance of social factors in addiction ^50^ and findings from this work, very little empirical evidence exists that explain how social cognition is impacted in nicotine dependent subjects at either the neural or the computational level. In one study, Chung and colleagues used a peer influence paradigm and fMRI ^51^ in adolescents; they found that substance naïve teens showed enhanced vmPFC activations towards safe choices made by peers, compared to teens who had used substances ^52^. This result suggests that substance use might be associated with reduced ability to distinguish benevolent vs. malevolent social signals. Our work is consistent with this study and expands our knowledge about the social brain in addition in demonstrating that substance use can be associated with both reduced ability to encode social value signals, and impairment in learning from social signals or using them to exert control during interpersonal interactions.

Limitations of the current study include a small sample size and low representation of females (due to higher tobacco use in males) of the fMRI study. Although the male-to-female ratio was less biased in the online sample (43% females), it is important to note that sex imbalances are often observed in the general population ^53,54^. As such, further larger-scale studies are needed to address the potential sex differences in nicotine addiction-related neural mechanisms and to provide more conclusive insights. Furthermore, although we were able to demonstrate group differences between smokers and controls, we did not find meaningful association between task behaviors and clinical measures capturing subjects’ severity of tobacco use or craving. Futures studies may investigate the relationship between deprivation level and task-based measures by systematically manipulating participants’ abstinence.

In conclusion, our findings suggest that under-estimation of the future consequences of their choices may be a key feature of nicotine-dependent humans and contribute to their inability to exert control in social settings. This serves as a plausible neurocomputational account for the social cognitive deficits observed in this population.

## Methods

### Participants

#### In-person study:

The fMRI study was approved by the Institutional Review Board (IRB) of the University of Texas at Dallas and the University of the Texas Southwestern Medical Center (where SN, MH, VFG, and XG worked and collected this dataset). All participants were recruited from the Dallas-Forth Worth metropolitan area through advertisements and flyers. All participants provided written informed consent before participating in the study and were compensated for their time. The criteria for in-person smoker recruitment included participants who smoked more than 10 cigarettes daily for at least a year and were fluent in English. All candidate participants underwent Structured Clinical Interview for DSM Disorders (SCID) – substance use disorder (SUD) module. For all participants, the exclusioncriteria were any major medical, neurological, or psychiatric conditions; any incompatibility with MRI safety (e.g. metal implants); and dependence on substances other than nicotine and alcohol (smokers) or any substance dependance (non-smokers). In the final sample, smokers had a mean daily consumption of 18 cigarettes, and mean baseline carbon monoxide (CO) level of 15.59 (SD: 8.89) parts per million (ppm). A total of 25 non-smoking and 17 nicotine-smoking participants were included in the final fMRI sample (**Table S1**).

#### Online study:

The online study was approved by the IRB of the Icahn School of Medicine at Mount Sinai. We recruited U.S.-based participants from the online subject pool Prolific (http://prolific.co). Here, we included a wider range of smokers (smoked at least one cigarette per week) to examine if findings from the in-person sample whose nicotine addiction was severe might generalize to a more representative sample of smokers with a wider range of nicotine dependence levels. Smokers with self-reported medical or psychiatric diagnosis were excluded. The final online smoker sample (n = 72) had a mean daily tobacco consumption of 9.34 and had a mean craving score of 64.51 out of 100. All participants provided online consent before participating in the study and were compensated for their time. The criteria for online non-smokers included zero tobacco consumption, no cravings for tobacco in the past week, and no major medical or psychiatric diagnosis. A total of 147 online participants met these criteria for non-smokers and were matched with online smokers for sex, age, education, and handedness (**Table S2**).

### Study Procedure

For the in-person study, all candidate participants underwent Structured Clinical Interview for DSM Disorders (SCID) – substance use disorder module, which was used to determine if they had nicotine addiction and/or other comorbid substance use disorder. For smokers, we also measured their exhaled CO levels using a smokerylzer (Covita Smokerlyzer) and administered a battery of questionnaires on their demographics and smoking habits. Specifically, the Shiffman-Jarvik Withdrawal Scale ^55^ was used to assess participants’ craving and withdrawal symptoms. Non-smokers completed a survey of their demographics. Participants were then asked to withdraw from smoking 12 hours prior to the next scheduled visit.

On the day of scanning, CO levels were re-evaluated. Participants played a two-party exchange task in a Phillips 3T MRI. A Phillips 3T MRI scanner was used to obtain anatomical and functional images of participants completing the task. High-resolution structural images were collected using a multi-echo MP-RAGE sequence with the following parameters: TR/TE/TI = 2300/2.74/900 ms, flip angle = 8°, FOV = 256x256 mm, Slab thickness = 176, Voxel size = 1x1x1 mm, Number of echos = 4, Pixel bandwidth = 650 Hz, Total scan time = 6 min. These structural scans were used for alignmemnt of images. fMRI scans were obtained by setting repetition time (TR) to 2000 ms, echo time (TE) to 25ms, voxel size to 3.4 mm × 3.4 mm × 4.0 mm, flip angle to 90°, and slice number to 37.

For the online study, after participants consented for research, they completed a battery of surveys that assessed demographics, mental health, and substance use as well as the social controllability task, as described below.

### Social Controllability Task

All participants completed a two-party exchange task ^1^ adapted from the ultimatum game in which simulated partners proposed how to divide a sum of $20 and participants decided whether to accept or reject the offer. If the participant accepted the offer, both the responder (participant) and the proposer received the proposed amount as is. If the participant rejected the offer, neither party received a reward. Offers were always disadvantageous to the participant/responder (<=$9) and the initial offer was always $5 (“indifference point”).

Importantly, we modified the game so that participants could influence their partner's future monetary proposal using their current actions ^1^ (Fig. 1). Specifically, if participants rejected the current offer, the next offer would increase by $0, $1 or $2 with a 1/3 probability for each option; and if they accepted the current offer, the next one would decrease by $0, $1 or $2 with a 1/3 probability for each (Fig. 1B). We also included a typical ultimatum game block in which the offer was randomly drawn from normal distribution with a mean of $5 and subjects’ current choice to accept or reject the offer had no influence over the future offers (“uncontrollable” condition; see **Supplementary Material Figure S1** ).

Subjects were told that they were playing with members of two different teams and were not given information regarding how the two teams might differ. The order of the conditions was counterbalanced. The original task with healthy controls included 40 trials per condition and smokers played a slightly shorter version of 30 trials that were shown to generate similar results ^1^. Nevertheless, to match the task length between smokers and non-smokers, only the first 30 trials from healthy control data were included in the analyses. After completing the task, subjects were asked to rate their perceived influence over their partners’ offers in each condition using a scale from 0 to 100 (“perceived controllability).

### Computational Modeling of Choice Behavior

The *forward thinking (FT) value,* or mentally projected total value of an action taken at the ith trial, $v{|}_{a_{i}}$, ($a_{i},acceptanceorrejection$)is estimated in an n-step forward thinking model, which considers various planning horizons given hypothetical future actions. Here the FT value $v{|}_{a_{i}}$ takes into account both current and future utilities of a choice.


$$v{|}_{a_{i}}=U\;(r_{i},f_{i})+\sum_{j=1}^{n}\gamma^{j}\times U\left(\hat{E}\;(r_{i+j}|\;a_{i},a_{-i+1},\ldots a_{-i+j}),f_{i}\right)$$

C*urrent utility* $U\;(r_{i},f_{i})$ is a function of reward $r_{i}$ and internal norm $f_{i}$ at the ith trial, defined as follows:

$$U\;(r_{i},f_{i})=\left\{\begin{array}{c} r_{i}-\alpha \max\;[f_{i}-r_{i},0]\;\text{if}r_{i}>0\;(accepted)\\ 0\text{if}r_{i}\;=\;0\;(rejected) \end{array}\right\}$$

The degree of aversion or sensitivity to norm violation at the individual level is captured by α(0≤α≤1) ^56^. Internal norm $f_{i}$ is a measure of subjective norm, or one’s trial-by-trial expectation of the offer. Here, we assumed that participants update their internal representation of the norm from trial to trial using the Rescorla-Wagner learning model based on our previous work ^57^ and that the initial norm $f_{0}$ varies from individual to individual with a range of [$0, $20] ^42^.


$$f_{i}=f_{i-1}+\epsilon \left(s_{i}-f_{i-1}\right)$$

Here the learning rate ε(0≤ε≤1) represents how fast one updates expectation of the offer based on the *norm prediction error* (nPE), defined as $\left(s_{i}-f_{i-1}\right)$.

*Future utility* is described as the summed utility of the mentally simulated future actions $a_{-}$ discounted by γ, the temporal discounting factor. Similar to our previous work, we fixed γ at 0.8, the mean value measured in a larger cohort to control for collinearity with our parameter of interest $\delta^{1}$. $\hat{E}$ is the subject’s mentally simulated future split. Importantly, our parameter of interest δ represents how much (in dollar amount) a participant thought their action changed the partner’s proposed split at a future trial, $a_{-k}$, in the following manner:

E^(sk+1)={sk+δifakora−k=0max(sk−δ,1)ifakora−k=1}

a_k={1ifU(E^(sk),fk)>00otherwise}

Critically, δ represents a subject’s mentally *estimated influence* of their current action on the subsequent offer (in dollar amount, ranging from −$2≤δ≤$2)). The simulated future action $a_{-k}$ of accepting an offer is determined by the subjective utility of the following rewards $U\left(\hat{E}\;(s_{k})\;,\;f_{k}\right)$. In the event that the simulated chosen action is to accept the offer $\left(a_{-k}=1\right)$, the hypothetical next offer $\hat{E}\;(s_{k+1})$ decreases by the *estimated influence* parameter δ(−$2≤δ≤$2). In the event that the simulated chosen action is to reject the offer $\left(a_{-k}=0\right)$ the hypothetical next offer $\hat{E}\;(s_{k+1})$ increases by δ. Here δ is applied symmetrically to acceptance and rejection, also similar to our previous work ^1,58^.

Action selection was based on the difference between the total projected value of accepting an offer ($v{|}_{a_{i}=1}$) and the total projected value of rejecting an offer ($v{|}_{a_{i}=0}$).

$${\Delta Q}_{i}=v{|}_{a_{i}=1}-v{|}_{a_{i}=0}$$

${\Delta Q}_{i}$ in turn influences the probability of choosing an action in a softmax function:

$$P_{i}\;(a_{i}=1)=\frac{e^{{\beta \Delta Q}_{i}}}{1+e^{{\beta \Delta Q}_{i}}}$$

Behavioral responses were fitted into five models, each incorporating different planning horizons: 0-step, 1-step, 2-step, 3-step, 4-step. The 0-step model represents a standalone norm learning model and excludes any forward thinking. The other four models assume that an agent simulates the value of an action by considering both current and future values, all based upon the estimated levels of controllability of the social environment. We additionally fitted a model-free reinforcement learning model which only considers cached values (see **Supplemental Information** (S2) for details). The best fitting model was chosen based on both Deviation Information Criteria (DIC) (where a smaller index indicates both higher model evidence and lower model complexity; see **Table S3**) and the recoverability of model parameters (see **Table S4-S5**).

Individual choices from middle trials (trials 6–25) were used for model fitting. The first 5 trials were excluded from all participants’ data to allow behavior to stabilize after participants explored the contingencies of the task in these initial trials. The last 5 trials from the smokers’ responses were also excluded given that there was less incentive to reject offers closer to the end of the game ^38^. Finally, the last 15 trials from the non-smokers’ responses were excluded in order to maintain trial number consistent to that of the smokers during analysis.

### fMRI data analysis

The functional scans were analyzed using the statistical parametric mapping software package (SPM12, Wellcome Department of Imaging Neuroscience; www.fil.ion.ucl.ac.uk/spm). First, we preprocessed the images by implementing time correction, co-registration, and normalization with resampled voxel size of 2mm × 2mm × 2mm and smoothing with an 8mm Gaussian kernel. After preprocessing, two general linear models (GLMs) were constructed using SPM12 to examine the neural correlates of 1) forward thinking value and 2) norm prediction errors (PEs). The following event regressors were included: 1) offer onset, 2) choice submission, 3) outcome onset, and 4) perceived controllability rating.

Importantly, we specified a parametric modulator of FT value, the forward projected choice value from the 2-step model, normalized at the individual level, at the onset of choice submission. A separate GLM was conducted in which the learning signal nPE replaced the total choice values as the parametric regressor. In both GLMs, six motion parameters were included as covariates. Following individual model estimation at the 1st (subject) level, contrast images representing either total choice value or norm PE were entered into an ANOVA test to compare neural differences between smokers and non-smokers ($P_{FDR}<0.05$ and k>50).

We used the MarsBar toolbox ^59^ to conduct region of interest (ROI) analyses. Beta values representing choice value-related activations were extracted from an 8-mm radius sphere of the vmPFC using coordinates [−2, 50, −2] from an independent study ^21^. Beta values representing norm PE were extracted at a coordinate of the midbrain [−4, −26, −11] on an 8-mm radius sphere, from an independent study ^25^.

## Figures and Tables

**Table 1:** Parameter estimates from the 2-step forward thinking model. Mean (SD) of parameters estimated in the model include inverse temperature, sensitivity to norm violation, initial norm, adaptation rate and estimated influence (parameter of interest). Statistics for the fMRI sample are obtained through a two-sample t-test, while the online sample utilizes a non-parametric bootstrapping test.

	Inverse temperature	Sensitivity to norm violation	Initial norm	Adaptation rate	Estimated influence
	β	α	μ	ε	δ
**fMRI Sample**					
Non-smokers	8.814 (8.464)	0.687 (0.313)	8.342 (7.555)	0.171 (0.160)	1.396 (0.654)
Smokers	9.172 (7.662)	0.699 (0.411)	10.517(7.478)	0.284 (0.338)	0.352 (1.544)
t-value	0.14	0.105	0.92	1.454	−3.018
p-value	0.445	0.458	0.182	0.077	0.002**
**Online Sample**					
Non-smokers	9.032(8.498)	0.754(0.238)	8.418(6.994)	0.336(0.295)	1.351(0.833)
Smokers	9.206(8.737)	0.743(0.311)	9.871(7.883)	0.306(0.315)	1.119(1.016)
t-value	−0.140	0.269	−1.328	0.679	1.677
p-value	0.438	0.388	0.093	0.253	0.045*
